# Dietary Patterns in Pregnancy in New Zealand—Influence of Maternal Socio-Demographic, Health and Lifestyle Factors

**DOI:** 10.3390/nu8050300

**Published:** 2016-05-19

**Authors:** Clare R. Wall, Cheryl S. Gammon, Dinusha K. Bandara, Cameron C. Grant, Polly E. Atatoa Carr, Susan M. B. Morton

**Affiliations:** 1 Centre for Longitudinal Research—He Ara ki Mua, University of Auckland, Auckland 1072, New Zealand; c.gammon@auckland.ac.nz (C.S.G.); d.bandara@auckland.ac.nz (D.K.B.); cc.grant@auckland.ac.nz (C.C.G.); p.atatoacarr@auckland.ac.nz (P.E.A.C.); s.morton@auckland.ac.nz (S.M.B.M.); 2 Discipline of Nutrition and Dietetics, School of Medical Sciences, University of Auckland, Auckland 1072, New Zealand; 3 Department of Paediatrics, Child & Youth Health, University of Auckland, Auckland 1072, New Zealand; 4 Starship Children’s Hospital, Auckland District Health Board, Auckland 1148, New Zealand; 5 Child Health, Waikato District Health Board, Hamilton 3204, New Zealand

**Keywords:** dietary patterns, pregnancy, principal component analysis, ethnicity

## Abstract

Exploration of dietary pattern associations within a multi-ethnic society context has been limited. We aimed to describe dietary patterns of 5664 pregnant women from the *Growing Up in New Zealand* study, and investigate associations between these patterns and maternal socio-demographic, place of birth, health and lifestyle factors. Participants completed a food frequency questionnaire prior to the birth of their child. Principal components analysis was used to extract dietary patterns and multivariable analyses used to determine associations. Four dietary components were extracted. Higher scores on, ‘Junk’ and ‘Traditional/White bread’, were associated with decreasing age, lower educational levels, being of Pacific or Māori ethnicity and smoking. Higher scores on, ‘Health conscious’ and ‘Fusion/Protein’, were associated with increasing age, better self-rated health, lower pre-pregnancy body mass index (BMI) and not smoking. Higher scores on ‘Junk’ and ‘Health conscious’ were associated with being born in New Zealand (NZ), whereas higher scores on ‘Fusion/Protein’ was associated with being born outside NZ and being of non-European ethnicity, particularly Asian. High scores on the ‘Health conscious’ dietary pattern showed the highest odds of adherence to the pregnancy dietary guidelines. In this cohort of pregnant women different dietary patterns were associated with migration, ethnicity, socio-demographic characteristics, health behaviors and adherence to dietary guidelines.

## 1. Introduction

A woman’s diet and lifestyle before and during pregnancy can positively or negatively influence the health of both mother and child [1]. We have recently described the poor adherence of 5664 pregnant women in New Zealand (NZ) to the National Food and Nutrition Guidelines for Healthy Pregnant Women using conventional dietary analyses, with only 3% meeting the recommendations for all four food (fruit and vegetables, breads and cereals, milk and milk products and lean meat, meat alternatives and eggs) groups [2]. Similarly, in a recent Canadian study involving 2313 pregnant women, only 3.5% were found to consume the recommended number of servings for all four food groups [3].

The research which has informed guidelines such as these, for pregnant women, has largely utilized conventional dietary analysis which has concentrated on exploring associations between single nutrients or foods and health outcomes. This narrow focus fails to capture the cumulative and interactive effects of multiple components of diet in predicting health outcomes [4]. Further, food is consumed in various combinations which are influenced by many factors, including culture and food availability [5]. Therefore, there has been a shift to utilizing dietary pattern analysis, which captures overall diet quality and variety, to inform public health recommendations [4,6]. Interestingly, although there a number of approaches to dietary pattern assessment, including use of an a priori index or a data-driven approach, consistency has been reported in what are described as healthy and unhealthy dietary patterns [7]. 

Principal components analysis (PCA) is a commonly used, well established data-driven method of deriving dietary patterns [4,8] and has been employed in the exploration of diet during pregnancy in a number of studies. These studies have described the association of specific dietary patterns to socio-demographic and lifestyle factors [9,10,11] and have also been used to describe associations with birth outcome and maternal health [8,12,13,14,15,16,17]. However, to date, there has been limited exploration of dietary pattern associations within a multi-ethnic society context. This is in spite of the 21st century having seen unprecedented levels of international migration, and ethnic diversity becoming a demographic feature of many countries [18]. Further, the implications of these relationships have not been thought about in relation to current national nutrition guidelines for pregnant women.

Utilizing maternal dietary data collected from the longitudinal *Growing Up in New Zealand* study, the aim of our study was to describe the dietary patterns of pregnant women living in NZ and to investigate associations between these patterns and their socio-demographic, health and lifestyle characteristics, including ethnicity. In addition, we wanted to examine the associations between each maternal dietary pattern and adherence to the food and nutrition guidelines for the core food groups.

## 2. Materials and Methods

### 2.1. Study Population

The opportunity to complete our study was created by the recruitment of a large ethnically diverse sample of pregnant women to create NZ’s contemporary child cohort study *Growing Up in New Zealand* (www.growingup.co.nz).

Eligibility for enrolment into *Growing Up in New Zealand* was defined by having an estimated delivery date between 25 April 2009 and 25 March 2010, and residence in a geographically defined region of NZ selected for its ethnic, socio-economic and urban/rural diversity. There were no other inclusion or exclusion criteria [19]. Eleven percent of the births in NZ over the recruitment period were enrolled. Birth parameters for the cohort were broadly generalizable to all births in NZ from 2007 to 2010 [20]. Ethical approval was obtained from the Ministry of Health Northern Y Regional Ethics Committee. Written informed consent was obtained from all participating women.

### 2.2. Assessments

#### 2.2.1. Dietary Intake and Dietary Patterns

The data we used in this study to describe diet during pregnancy were gathered using a semi-quantitative 44 item food frequency questionnaire (FFQ) of dietary intake for the previous four weeks (see—www.growingup.co.nz). This was administered as part of the enrolment interview, which was most often conducted during the third trimester. In order to minimize recall bias we only included data from the women who completed the interview prior to the birth of their child (*n* = 5664). The purpose of the antenatal FFQ used in *Growing Up in New Zealand* was to describe the frequency of consumption of the four core food groups (as defined in the Ministry of Health guideline statements for healthy pregnant women): fruits and vegetables, breads and cereals, milk and milk products and lean meat, meat alternatives and eggs, and also foods likely to be high in fats, sugars and/or salt. The Ministry of Health recommends limiting the intake of foods and drinks that are high in fat (especially saturated fat), salt and/or sugar [21].

Within the FFQ, foods were grouped under subheadings: (1) fruits; (2) vegetables; (3) dairy products including milk, cheese, and yoghurt; (4) bread, rice, pasta, cereal, cakes and biscuits; (5) meat, processed meats, seafood, meat alternatives and eggs; (6) spreads including butter, margarine, jam, Nutella™, and peanut butter; (7) soft drinks, snacks, confectionary and sweets, with some additional specific questions asked about foods likely to be high in fats, sugars and/or salt. Frequency of intake of individual foods was described by categories increasing from not at all or less than once a month, less than once a month to a number of times per month, week or day. Show cards were used to illustrate a standard portion size.

Comprehensive training in administration of the FFQ was provided. This included piloting of the antenatal questionnaire (including the FFQ) to a group of 200 ethnically diverse pregnant women enrolled approximately six months earlier than the main cohort to assess the feasibility and acceptability of the questionnaire [22].

#### 2.2.2. Covariates

The antenatal enrolment interview also included questions on factors that allowed us to describe maternal socio-demographic, health and lifestyle characteristics of participants and/or other factors previously identified in the literature for their potential influence on dietary intake during pregnancy.

Detailed information gathered on self-prioritized ethnicity was coded to give six Level 1 categories following Statistics New Zealand’s coding criteria: (i) European; (ii) Māori; (iii) Pacific Peoples; (iv) Asian; (v) Middle Eastern/Latin American/African (MELAA) and (vi) Other ethnicity. Where women with multiple ethnicities did not provide a self-prioritized main ethnicity, and a single ethnic group was required for modelling purposes, an external prioritization was employed (as utilized by Statistics New Zealand from 1991 until 2004) [23]. Due to the small numbers constituting the MELAA and Other ethnicity groups, for the purposes of this analysis, the two groups were combined under the category ‘Other’. 

Household deprivation was described using the NZ Index of Deprivation (NZDep06) which is created using data from the 2006 National Census around nine socioeconomic indicators: home ownership, single-parent family, household income, means-tested benefits, qualifications, employment, access to a car, access to a telephone and household crowding. The generated index score, averaged for a population of a geo-coded address area, has scores ranging from 1 (least deprived 10 percent) to 10 (most deprived 10 percent) [24]. For this analysis these scores are grouped as quintiles. 

Self-reported measures of pre-pregnancy height and weight allowed pre-pregnancy body mass index (BMI) to be calculated. Although it is recognized that self-reported BMI may result in some underestimation of BMI, it is considered to produce minimal bias when used in epidemiological studies to examine associations [25].

The maternal health characteristics included were a pre-pregnancy self-rating of health (poor/fair through to excellent; see Table 1), the degree of nausea/sickness after the first trimester and an assessment of antenatal depression risk. Depression risk was assessed using the Edinburgh Postnatal Depression Scale (EPDS), with a score of 13 or greater indicating that a women is likely to be suffering from symptoms of depression [26].

Finally, lifestyle characteristics considered included folic acid supplementation use, smoking and alcohol consumption, physical activity prior and during pregnancy, and whether actively dieting pre-pregnancy. Physical activity was assessed using questions from the International Physical Activity Questionnaire (IPAQ). Participants were asked about intensity (moderate or vigorous), duration (<30 min, 30–60 min, >60 min) and frequency (days per week) of activity [27]. To be classified as participating in moderate/vigorous activity women had to have engaged in moderate activity for at least 30 min for at least five out of seven days or vigorous activity for at least 30 min on at least two out of seven days. 

### 2.3. Statistical Analysis

We identified dietary patterns by placing all 44 standardized FFQ food items into a PCA and performing a varimax (orthogonal) rotation using PROC FACTOR in Statistical Analysis Systems (SAS). Rotating the factors orthogonally results in uncorrelated factors which can all be independently included in regression analyses [28]. We excluded from the PCA those with more than 10 missing dietary items (three women).

The number of patterns (components) identified was based on identification of a break point in the scree plot [29] and interpretation of the factor loading matrix after varimax rotation. Factor loadings are the coefficients defining the patterns and describe the correlations of each food item with that pattern. Food items with factor loadings of 0.3 or greater were considered to have a strong association with the identified pattern [30]. Each dietary pattern was labelled with the most appropriate term which best described the underlying pattern and were consistent with labelling schemes from previous studies of diet that have used PCA. Summary scores were then created for each woman for each dietary pattern (with a mean of 0.0 and a standard deviation (SD) of 1.0), with a higher score indicating stronger adherence to that dietary pattern.

Associations of maternal socio-demographic, health and lifestyle factors (age, pre-pregnancy BMI, self-prioritized ethnicity, place of birth, education, socioeconomic deprivation, pregnancy planning, parity, lead maternity carer (LMC) type, season of interview, maternal self-rating of health, nausea/sickness after 1st trimester, EPDS score, folic acid supplement intake, pre/during pregnancy smoking patterns, pre/during pregnancy alcohol consumption, physical activity, and actively dieting pre-pregnancy), with dietary patterns were investigated using univariable analysis (data not shown) and multivariable analyses, presented as parameter estimates (95% CI).

We have previously reported the cohort adherence to the NZ food and nutrition guidelines for pregnant women [2]. To determine the association between dietary patterns and adherence to nutritional guidelines (adherence to vegetables and fruits, adherence to breads and cereals, adherence to milk and milk products and adherence to lean meat, meat alternatives and eggs), multivariable logistic regression were carried out. These analyses were controlled for maternal socio-demographic, health and lifestyle factors and presented as OR (95% CI) related to 1 SD change in pattern score. Finally, logistic regression modelling was also performed to determine the association between dietary patterns and adherence to the number of guidelines.

All analyses were conducted using SAS software (version 9.3, SAS Institute, Cary, NC, USA).

## 3. Results

### 3.1. Sample Demographics

The characteristics of the *Growing Up in New Zealand* cohort have been described previously [19,20]. The maternal socio-demographic, health and lifestyle characteristics for the 5664 respondents whom antenatal dietary data were available are presented in Table 1. 

The median (range) age in years was 31 (15 to 47), with more than half in the 30–39 year age range and pre-pregnancy BMI was 23.8 (13–67) kg/m^2^. The self-prioritized ethnicity breakdown was European (56%), Māori (13%), Pacific Peoples (13%), Asian (14%), and Other ethnicities (4%), with nearly 35% of the women born outside NZ.

Ten percent of women self-rated their health as poor, and 12% of women were reported to have a score ≥13 on the EPDS. One half of the 21% of women who were smoking before pregnancy stopped smoking during the pregnancy. Twenty-seven percent of the women were non-alcohol drinkers and 44% of women who were alcohol drinkers gave up during pregnancy, while 29% of women continued drinking at some stage during their pregnancy.

### 3.2. Dietary Components Identified

Four distinct dietary patterns were extracted that best describe the dietary patterns of food intake during their third trimester of pregnancy. A total of 23.4% of the variability in food intake was explained by these four components, with the first explaining 7.7%, followed by 5.6%, 5.4% and 4.8%, respectively. Table S1 shows the factor loading obtained from the PCA.

**Figure 1:**
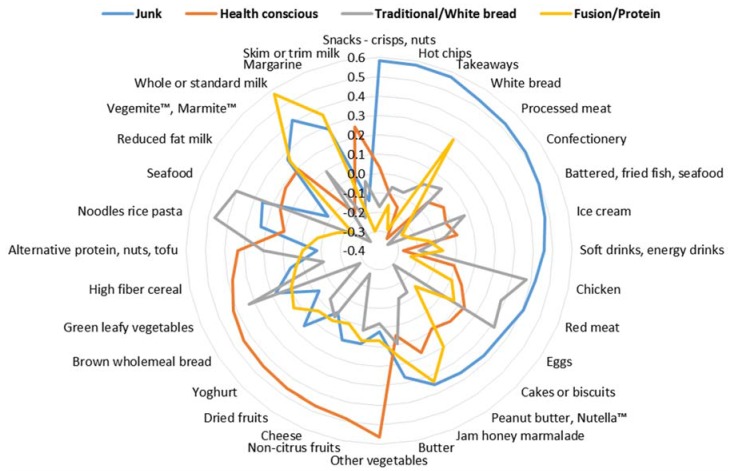
Factor loadings after varimax rotation on identified dietary patterns of food groups retained. Not shown are food items with values less than 0.3 (fruit juices, other cereal, citrus fruits, butter and margarine blend, high fibre white bread, processed fish, other bread, other milk, plant sterol margarine, soy milk, lite or reduced fat margarine, soft drinks sugar free).

The four components were labelled ‘Junk’, ‘Health conscious’, ‘Traditional/White bread’ and ‘Fusion/Protein’. The first component was described as ‘Junk’ because of the high loadings of confectionary, snacks, takeaways, hot chips, processed meats, soft and energy drinks, battered fried fish or seafood, ice-cream and cakes or biscuits. The second component was described as ‘Health conscious’ because of the high loadings of vegetables, cheese, brown whole meal bread, non-citrus fruits, yoghurt, dried fruits, high fiber cereal, and Vegemite™ or Marmite™. The third component was described as ‘Traditional/White bread’ because of the high factor loadings for whole or standard milk, white bread, margarine, jam honey marmalade, peanut butter, Nutella™ and low fiber and/or high sugar cereals. The fourth component was described as ‘Fusion/Protein’ because of the high factor loadings for noodles rice pasta, seafood, chicken, green leafy vegetables, eggs and red meat (Figure 1).

### 3.3. Associations with Maternal Socio-Demographic, Health and Lifestyle Factors

Dietary pattern scores were differently associated with nearly all the maternal socio-demographic, health and lifestyle characteristics. Table 2 presents the parameter estimates (95% CI) from the multivariable analysis.

The ‘Junk’ dietary pattern scores were positively associated with decreasing maternal age, being Māori or Pacific, lower educational levels, women rating their health as poor/fair or good, having an EPDS score ≥13 and actively dieting before-pregnancy. Compared to non-smokers and non-drinkers this pattern was also positively associated with stopping drinking and continuing to smoke and drink during pregnancy. The ‘Junk’ dietary pattern scores were negatively associated with women being born outside NZ, being of Asian ethnicity and taking folic acid supplementation before and during pregnancy.

The ‘Health conscious’ dietary pattern scores were positively associated with being over 40 years in age and taking folic acid supplementation before and during pregnancy. Compared to women who did no exercise (defined as moderate or vigorous activity) before and during pregnancy, this pattern was positively associated with exercise before and during pregnancy. The ‘Health conscious’ dietary pattern scores were negatively associated with decreasing maternal age, increasing pre-pregnancy BMI, being of Asian or ‘Other’ ethnicity, women being born outside NZ, having lower educational levels, rating their pre-pregnancy maternal health as poor/fair or good compared to very good, having moderate nausea (and occasional sickness) after the first trimester compared to mild nausea only. Compared to non-smokers, this pattern was negatively associated with continuing to smoke during pregnancy.

The ‘Traditional/White bread’ dietary pattern scores were positively associated with decreasing maternal age, being of Māori or Pacific ethnicity, lower educational levels and a multiparous birth. Compared to non-smokers, this pattern was positively associated with stopping smoking and continuing to smoke during pregnancy. Depending on the season of interview a positive association was observed with this dietary pattern and winter, compared to summer. The ‘Traditional/White bread’ dietary pattern scores were negatively associated with decreasing socioeconomic deprivation level, a planned pregnancy, taking folic acid supplementation before and during pregnancy, and actively dieting pre-pregnancy. Compared to non-drinkers this pattern was negatively associated with stopping drinking and continuing to drink during pregnancy. Compared to women who did no exercise before and during pregnancy, this pattern was negatively associated with no exercise during pregnancy.

The ‘Fusion/Protein’ dietary pattern scores were positively associated with being over 40 years in age, of non-European ethnicity, and in particular of Asian ethnicity, women being born outside NZ, and women rating their pre-pregnancy maternal health status as excellent compared to good. Compared to women who did no exercise before and during pregnancy, this pattern was positively associated with exercise before and during pregnancy. The ‘Fusion/Protein’ dietary pattern scores were negatively associated with decreasing maternal age, increasing pre-pregnancy BMI, a planned pregnancy, primiparous birth, rating their pre-pregnancy maternal health as poor/fair and taking folic acid supplementation before and during pregnancy. Compared to non-smokers, this pattern was negatively associated both stopping smoking and continuing to smoke during pregnancy.

### 3.4. Associations with Adherence to Dietary Guideline Recommendations

Adjusted multivariable associations between the four dietary patterns and adherence to dietary guideline recommendations for the number of daily servings from each of the four main food groups are presented in Table 3. The unadjusted associations are presented in supplementary materials Table S2.

Higher scores on both the ‘Health conscious’ OR (per 1 SD increase) 6.37 (95% CI; 5.29, 7.67) and ‘Fusion/Protein’ OR 4.79 (4.05, 5.65) dietary patterns were significantly associated with an increased odds of being adherent to the recommended guideline for the number of fruit and vegetable servings, whereas higher scores on the ‘Junk’ OR 0.56 (0.48, 0.65) dietary pattern were significantly associated with decreased odds. Higher scores on ‘Fusion/Protein’ OR 14.3 (11.5, 17.8) dietary pattern were significantly associated with increased odds of being adherent to the recommended guideline for the number of lean meat, meat alternatives and eggs servings, but was significantly associated with decreased odds, OR 0.56 (0.46, 0.67), for the number of breads and cereals servings. Neither the ‘Health conscious’ or ‘Traditional/White bread’ dietary patterns were significantly associated with being adherent to the recommended guideline for the number of lean meat, meat alternatives and eggs servings.

Table 4 presents the adjusted OR (95% CI) per 1.0 SD on the dietary pattern score for the multivariable analysis of the associations between each maternal dietary pattern and the number of food group guidelines for which maternal diet was adherent.

Women with the highest scores on the ‘Health conscious’ OR 19.2 (15.6, 23.6) dietary pattern showed the highest odds of being adherent to meeting two to four of the Ministry of Health’s Food and Nutrition Guidelines; This was followed by women with high scores on the ‘Fusion/Protein’ and ‘Traditional/White bread’ dietary patterns with women with high scores on the ‘Junk’ OR 1.66 (1.41, 1.96) dietary pattern having the lowest odds ratio of meeting two-four guidelines.

## 4. Discussion

In this cohort of pregnant NZ women (*n* = 5664), data collected by a FFQ during their third trimester of pregnancy and analyzed by PCA revealed four distinct dietary patterns. Based on the foods which they contained, two more-healthy dietary patterns, labelled ‘Health conscious’ and ‘Fusion/Protein’ and two less-healthy dietary patterns, ‘Junk’ and ‘Traditional/White bread’. Clear associations could be identified between having higher scores for these patterns and a range of maternal socio-demographic, health and lifestyle factors and adherence to the food and nutrition guidelines for the core food groups. Specifically, women with high scores on the ‘Health conscious’ dietary pattern were the most likely to adhere to healthy eating guideline while those while those with high scores on the ‘Fusion/Protein’ and ‘Traditional/White bread’ patterns had moderate levels of adherence and those with high scores on the ‘Junk’ dietary pattern were the least likely to adhere to the guidelines.

Principal components analysis is one of the main techniques employed to generate empirically derived dietary patterns. In PCA, dietary data most commonly collected through FFQ or dietary records are reduced into dietary patterns based on inter-correlations among individual items [4,28]. Despite differences in methodology, including the labelling of patterns and the varied range of foods eaten by different population groups, in most analyses a healthy dietary pattern, with high factor loadings for healthy food items such as fruit, legumes, fish and high fiber breads and cereals, and a less-healthy dietary pattern, with high factor loading for foods such as processed meats, refined grains and added sugar (usually processed sweet food such as biscuits, *etc.*) can be described. Other dietary patterns described often reflect the influence of geographic region or culture on dietary intake of a population [28]. 

The first two dietary patterns extracted in this study were ‘Junk’ and ‘Health conscious’. High scores on the ‘Junk’ dietary pattern were associated with younger maternal age and lower educational levels, being born in NZ and being of Pacific or Māori ethnicity, but not Asian, and unhealthful behaviors such as not taking folic acid supplementation, smoking, and alcohol consumption during pregnancy. High scores on this dietary pattern were also associated with increased risk of antenatal depression. In contrast, high scores on the ‘Health conscious’ dietary pattern were associated with higher maternal age and educational levels, a lower pre-pregnancy BMI and better self-rated health. Further, high scores on this pattern were associated with positive health behaviors including taking folic acid supplements and exercising before and during pregnancy and not smoking during pregnancy. As with the ‘Junk’ dietary pattern, higher scores on this pattern were also associated with being born in NZ and not being of being Asian ethnicity. Additionally, they were associated with not being in the category of ‘Other’ ethnicities.

The third pattern described in this study is the ‘Traditional/White bread’ dietary pattern (whole or standard milk, white bread and spreads and low fiber cereals) reflects foods associated with a ‘Traditional’ uncooked NZ breakfast. This pattern could also be considered nutrient poor, as could the ‘Junk’ food dietary pattern. Similarly it was associated with being of Pacific or Māori ethnicity, younger maternal age and lower educational levels, not taking folic acid supplements and smoking. However, scores on this pattern were not associated with alcohol use during pregnancy. It was the only pattern to be associated with socioeconomic status, with women with high scores more likely to live in areas of high deprivation, and it was associated with having an unplanned, multiparous birth. The final dietary pattern, ‘Fusion/Protein’, which combines elements of Asian cuisine with animal protein sources. High scores on this pattern were strongly associated with being of Asian ethnicity, followed by Pacific, Other and Māori and being born outside NZ and were associated with higher maternal age, a primiparous birth, better self-rated health, and some positive health behaviors including stopping smoking and exercising before and during pregnancy.

In general these findings are in-line with previous cohort studies which have examined associations between maternal dietary patterns and various socio-demographic factors. The Finnish Type 1 Diabetes Prediction and Prevention (DIPP) Nutrition study, Danish National Birth Cohort (DNBC), the British Avon Longitudinal Study of Parents and Children (ALSPAC) and Norwegian Mother and Child (NMC) studies all reported that their ‘Healthy’ dietary pattern was associated with higher age and education levels (not measured in DNBC), lower pre-pregnancy measure of BMI/weight (not measured in DIPP) and not smoking [9,11,13,14]. Whereas the cohorts’ ‘Unhealthy’ dietary patterns showed the reverse associations, *i.e.*, associations with decreasing age, lower education levels and smoking [9,11,13,14]. Of these studies, ALSPAC was the only cohort to include ethnicity as a variable, which classified subjects as white and non-white, but the non-white group comprised only 2.6% of the cohort. In a NZ case-control cohort of individuals born appropriate for gestational age and small for gestational age, the Auckland Birthweight Collaborative (ABC) study identified three dietary patterns: a ‘Junk’ dietary pattern, where high scores were found to be associated with being of Pacific or Māori ethnicity; a ‘Fusion’ dietary pattern, where high scores were associated with being of Asian, Māori and Pacific ethnicities and a ‘Traditional’ dietary pattern, although it differed quite markedly in food composition from our ‘Traditional/White bread’ dietary pattern [16].

Good nutrition during pregnancy, including appropriate supplement use, exercise and avoidance of alcohol and smoking are all key elements of a healthy lifestyle promoted to optimize both maternal health, including mental health, and optimal fetal growth and development [1,21]. In this cohort, poor adherence to the Ministry of Health’s Food and Nutrition Guidelines in Pregnancy has been reported, with only 3% meeting the recommendations for all four food groups [2]. This was in spite of nearly 72% of women reporting that they had changed their diet as a result of information received during their pregnancy [22].

When examining adherence to meeting between two and four of the Ministry of Health’s Food and Nutrition Guidelines for core food groups by dietary patterns, levels of adherence varied between the different patterns, with women who had high scores on the ‘Junk’ dietary pattern having the lowest odds of adherence, particularly for fruit and vegetables servings. In contrast, the women with high scores on the ‘Health conscious’ dietary pattern had the highest odds of meeting between two and four of the guidelines and meeting the guideline for fruit and vegetables servings but not the guideline for lean meat, meat alternatives and eggs servings. A different approach to testing the internal validity was used by the Southampton Women’s Survey and ALSPAC studies. Both examined the relationship between their maternal dietary patterns and nutrient intakes, as assessed by dietary records and in both studies, after adjusting for energy, the ‘Healthy’ dietary pattern showed positive associations with protein, fiber and nearly all micronutrients assessed [31,32].

The number and diversity of new immigrants to NZ, as has been seen in most developed countries, has meant both an increase in the size of other ethnic groups (other than Europeans) and the number of people who were born overseas [33]. Building on the findings of Thompson *et al*., which showed that different dietary patterns during pregnancy are associated with different ethnic groups, we have also shown that different dietary patterns are associated with women being born in or outside of NZ. The degree of dietary acculturation by migrants varies according to a range of cultural, environmental and social factors, but evidence suggests that the higher incidence of chronic diseases seen in some ethnic populations post-migration is a result of the adoption of increased portion sizes and the addition of less-healthy food components [34,35,36]. Given the implications for future maternal health of the adoption of a less healthy pattern, including an increase in susceptibility to diet-related health problems such as obesity, type 2 diabetes and cardiovascular disease [34,35,36], nutrition education initiatives aimed at newly arrived migrants which encourage the maintenance of healthy dietary patterns could have long-term benefits.

The strong association between being of Asian ethnicity and being born outside NZ and high scores on the ‘Fusion/Protein’ dietary pattern likely reflects the more recent immigration patterns of people from Asia to NZ [33]. Women with high scores on this pattern had the second highest odds of meeting between two and four of the Ministry of Health’s Food and Nutrition Guidelines for core food groups and some positive health behaviors, although taking folic acid supplementation was not one of them. We have also previously identified women of Asian ethnicity as one ethnic group that had lower odds of taking folic acid supplementation before pregnancy compared to NZ Europeans [37]. This group, therefore, is an example of where a more targeted promotion to encourage women to maintain their traditional dietary patterns, in conjunction with promoting folic acid supplementation, could improve the nutritional status of these women.

ALSPAC examined the association of the dietary patterns found from their cohort with depression and anxiety, using the EPDS and the Crown Crisp Experimental Index, respectively [11]. No significant findings were seen for depression, however, their confectionary pattern was found to be positively associated with being anxious. A recent meta-analysis by Lai *et al.* showed that a ‘Healthy’ dietary pattern was significantly associated with reduced depression risk in adults. Conversely, a ‘Western’ dietary pattern showed a trend to increasing risk of depression, with a lack of power being thought to be the reason significance was not reached [38]. The ‘Junk’ dietary pattern in this study was significantly associated with having an EPDS score ≥13, indicating the presence of symptoms of depression, whereas the ‘Healthy’ and ‘Asian/Fusion’ dietary patterns were associated with better self-rated health. It has been suggested that poor diet quality, in conjunction with increased nutrient requirements during the perinatal period, may increase a woman’s vulnerability to depressive states, with estimates that the prevalence of antenatal depression in pregnant women might be as high as 20% [39].

Strengths of this study include its prospective design, antenatal enrolment and the ethnic and socio-demographic diversity of the *Growing Up in New Zealand* cohort. The cohort’s close alignment with all NZ births between 2007 and 2010 means that data is generalizable to the NZ population. Further, the questionnaire, including the FFQ, was interviewer-administered and collected detailed information on a wide range of relevant factors. However, this also meant that it was necessary to limit the size of the FFQ to 44 items. We believe that this FFQ was sufficiently detailed to provide an analysis of adherence to dietary recommendations. Southampton researchers recently developed a 20-item FFQ for adherence to a ‘Prudent’ dietary pattern, which was found to correlate strongly (*r* = 0.94) with the original 100-item FFQ used in the Southampton Women’s Survey [40]. Nevertheless the collapsing of foods into larger groups to reduce the number of items in a FFQ can reduce the ability of a FFQ to differentiate individual food-choice differences [41]. This may have contributed to the relatively low (23.4%) degree of variance of food intake captured in this analysis; however this level still falls well within what other similar studies have reported. Although other cohort studies have used more comprehensive FFQs, the data on diet during pregnancy has either been collected retrospectively after birth [9,16] and/or have been self-completed [9,11,13,14]. Self-completed FFQs rely heavily on participants having a reasonable level of literacy [42].

Dietary data will continue to be collected for the children of the *Growing Up in New Zealand* cohort at various time points. This will allow the longitudinal tracking and examination of influences on the children’s diet over time, including the relationship with the mother’s dietary pattern during pregnancy and other maternal socio-demographic variables.

All women should be supported to optimize their nutrition and health behaviors during pregnancy. However, this and other studies have shown that higher scores on a healthy dietary pattern is associated with certain socio-demographic attributes including age and a higher level of education. These factors, in conjunction with the ethnic differences and the high level of self-reported dietary change seen in our study, suggest that some different approaches to promote behavioral change and more targeted nutrition programs may be required to improve the nutritional status of all pregnant NZ women.

## 5. Conclusions

The current NZ nutrition guidelines for pregnant women were developed to support and optimize nutrition and health behaviors during pregnancy. It is clear, however, that these guidelines are not being taken up by all women. Our analysis identified two more-healthy dietary patterns and two less-healthy dietary patterns and showed differences in associations between having a higher score for these patterns and ethnicity, place of birth and a range of socio-demographic factors, and to the degree of adherence to the current guidelines. These findings suggest new approaches need to be found to target and better engage different population sub-groups, so that these nuances can be addressed and the nutritional status of all pregnant NZ women improved. Thus improving our population’s health and reducing health inequalities that can begin even before birth.

## Figures and Tables

**Table 1:** Participant socio-demographic, health and lifestyle characteristics ^1^.

Characteristics	*n* (%)	Characteristics	*n* (%)
**Maternal socio-demographic characteristics**		Season of interview	
Age group (years)		Summer	922 (16.3)
<20	273 (4.8)	Autumn	1019 (18.0)
20–29	2195 (38.8)	Winter	1686 (29.8)
30–39	2992 (52.8)	Spring	2037 (36.0)
40 and over	204 (3.6)	**Maternal health characteristics**	
Pre-pregnancy BMI (kg/m^2^)		Pre-pregnancy maternal self-rating of health	
Median (Range)	23.8 (13.0–67.0)	Poor/Fair	556 (9.8)
Self-prioritised ethnicity		Good	1896 (33.5)
European	3168 (56.0)	Very good	2056 (36.3)
Māori	747 (13.2)	Excellent	1151 (20.3)
Pacific	726 (12.8)	Nausea/sickness—after 1st trimester	
Asian	802 (14.2)	No nausea	3617 (63.9)
Other	213 (3.8)	Mild nausea (nausea only)	1154 (20.4)
Place of birth		Mod. nausea (occasionally sick)	646 (11.4)
New Zealand	3703 (65.4)	Severe nausea (regularly sick)	244 (4.3)
Outside New Zealand	1961 (34.6)	EPDS score ^2^	
Education		No (<13)	4992 (88.1)
Primary	352 (6.2)	Yes (≥13)	672 (11.9)
Secondary	1326 (23.5)	**Maternal lifestyle characteristics**	
Tertiary	3975 (70.3)	Folic acid intake	
Socioeconomic deprivation		Never taken folic acid	800 (14.1)
1 to 2 (least deprived)	936 (16.5)	Before pregnancy	2277 (40.2)
3 to 4	1080 (19.1)	After pregnancy	2587 (45.7)
5 to 6	1009 (17.8)	Pre/during pregnancy smoking patterns	
7 to 8	1172 (20.7)	Continued smoking	544 (9.6)
9 to 10 (most deprived)	1465 (25.9)	Stopped smoking	550 (9.7)
Pregnancy planning		Non-smokers	4555 (80.6)
Planned	3488 (61.8)	Pre/during pregnancy alcohol consumption	
Unplanned	2156 (38.2)	Any drinking during	1649 (29.1)
Parity		Stopped drinking	2481 (43.9)
First child	2396 (42.3)	Non-drinkers	1528 (27.0)
Subsequent child	3268 (57.7)	Pre/during pregnancy physical activity	
LMC type		Mod/vigorous activity during	156 (2.8)
GP (Family doctor)	41 (0.7)	No mod/vigorous activity during	1354 (23.9)
Independent midwife	3649 (66.0)	Mod/vig. activity before & during	1894 (33.4)
Hospital midwife	818 (14.8)	No mod/vig. activity before & during	2260 (39.9)
Obstetrician	445 (8.1)	Actively dieting pre-pregnancy	
Shared care GP and midwife	247 (4.5)	Yes	1460 (25.8)
Combination of care	331 (6.0)	No	4201 (74.2)

^1^ Number of participants and percentages unless otherwise indicated, *n* 5664, missing values for each socio-demographic and lifestyle factor have not been included in the column %; ^2^ EPDS score of 13 or greater indicates that a women is likely to be suffering from symptoms of depression [26]; BMI, body mass index; LMC, lead maternity carer; EPDS, Edinburgh Postnatal Depression Scale.

**Table 2:** Association of each maternal dietary pattern with various maternal socio-demographic, health and lifestyle factors, adjusting for all other factors in the table (Parameter estimates (PE) and 95% confidence intervals (CI)).

	Junk			Health Conscious			Traditional/White Bread			Fusion/Protein		
	*p*	PE	95% CI	*p*	PE	95% CI	*p*	PE	95% CI	*p*	PE	95% CI
Age group (years)	<0.0001			<0.0001			<0.0001			<0.0001		
<20		0.56	0.41, 0.71		−0.38	−0.54, −0.22		0.48	0.34, 0.63		−0.12	−0.27, 0.03
20–29		0.12	0.07, 0.18		−0.13	−0.19, −0.07		0.19	0.13, 0.24		−0.12	−0.18, −0.06
30–39			Ref			Ref			Ref			Ref
40 and over		−0.15	−0.28, −0.02		0.15	0.01, −0.28		0.10	−0.03, 0.23		0.14	0.01, 0.27
Pre-pregnancy BMI (kg/m^2^)	0.271	−0.003	−0.0078, 0.0022	0.002	−0.008	−0.0135, −0.0030	0.420	−0.002	−0.0069, 0.0029	0.033	−0.006	−0.0105, −0.0004
Self-prioritized ethnicity	<0.0001			<0.0001			<0.0001			<0.0001		
European			Ref			Ref			Ref			Ref
Maori		0.21	0.12, 0.29		0.02	−0.07, 0.11		0.40	0.31, 0.48		0.12	0.04, 0.21
Pacific		0.25	0.14, 0.35		−0.04	−0.15, 0.07		0.56	0.46, 0.67		0.52	0.42, 0.63
Asian		−0.32	−0.41, −0.23		−0.60	−0.70, −0.51		0.09	0.00, 0.18		0.88	0.79, 0.98
Other		−0.13	−0.26, 0.00		−0.21	−0.34, −0.07		0.02	−0.11, 0.15		0.27	0.14, 0.40
Place of birth	0.006			0.001			0.948			<0.0001		
New Zealand			Ref			Ref			Ref			Ref
Outside New Zealand		−0.09	−0.16, −0.03		−0.12	−0.19, −0.05		0.00	−0.07, 0.06		0.18	0.11, 0.25
Education	0.034			<0.0001			0.005			0.339		
Primary		0.17	0.04, 0.30		−0.11	−0.24, 0.03		0.21	0.08, 0.33		−0.10	−0.23, 0.03
Secondary		0.01	−0.05, 0.07		−0.20	−0.26, −0.13		0.04	−0.02, 0.10		−0.01	−0.08, 0.05
Tertiary			Ref			Ref			Ref			Ref
Socioeconomic deprivation	0.750			0.103			<0.0001			0.336		
1 to 2 (least deprived)		−0.05	−0.14, 0.03		0.11	0.02, 0.20		−0.23	−0.32, −0.15		−0.07	−0.16, 0.01
3 to 4		−0.04	−0.12, 0.04		0.03	−0.05, 0.12		−0.19	−0.27, −0.11		−0.07	−0.15, 0.01
5 to 6		−0.05	−0.13, 0.03		0.06	−0.02, 0.15		−0.18	−0.26, −0.10		−0.02	−0.10, 0.06
7 to 8		−0.03	−0.11, 0.05		0.08	−0.01, 0.16		−0.13	−0.20, −0.05		−0.05	−0.13, 0.03
9 to 10 (most deprived)			Ref			Ref			Ref			Ref
Pregnancy planning	0.390			0.206			0.0002			0.034		
Planned		0.03	−0.03, 0.09		0.04	−0.02, 0.11		−0.12	−0.18, −0.06		−0.07	−0.13, −0.01
Unplanned			Ref			Ref			Ref			Ref
Parity	0.061			0.763			<0.0001			0.017		
First child			Ref			Ref			Ref			Ref
Subsequent child		0.05	0.00, 0.10		0.01	−0.05, 0.06		0.24	0.19, 0.30		−0.06	−0.12, −0.01
LMC type	0.046			0.174			0.056			0.841		
GP (Family doctor)		−0.13	−0.43, 0.17		−0.11	−0.42, 0.20		0.09	−0.21, 0.38		−0.08	−0.38, 0.23
Independent midwife			Ref			Ref			Ref			Ref
Hospital midwife		−0.02	−0.09, 0.05		−0.05	−0.12, 0.03		−0.01	−0.08, 0.06		0.05	−0.03, 0.12
Obstetrician		0.11	0.02, 0.20		−0.10	−0.19, 0.00		−0.14	−0.23, −0.06		0.00	−0.09, 0.09
Shared care GP & midwife		0.03	−0.10, 0.16		−0.11	−0.25, 0.02		−0.01	−0.14, 0.11		0.01	−0.12, 0.14
Combination of care		0.11	0.01, 0.22		0.00	−0.10, 0.11		−0.03	−0.13, 0.07		−0.02	−0.12, 0.09
Season of interview	0.470			0.939			0.0006			0.624		
Summer			Ref			Ref			Ref			Ref
Autumn		0.05	−0.04, 0.13		0.01	−0.08, 0.10		−0.06	−0.14, 0.02		0.02	−0.06, 0.10
Winter		0.01	−0.07, 0.08		−0.01	−0.09, 0.07		0.08	0.01, 0.15		−0.02	−0.10, 0.05
Spring		0.04	−0.03, 0.12		−0.01	−0.09, 0.06		−0.01	−0.08, 0.06		0.01	−0.06, 0.08
Maternal self-rating of health	0.013			<0.0001			0.844			0.0005		
Poor/Fair		0.13	0.03, 0.22		−0.20	−0.30, −0.09		0.02	−0.08, 0.11		−0.14	−0.24, −0.04
Good		0.08	0.02, 0.14		−0.10	−0.17, −0.04		0.01	−0.05, 0.07		−0.05	−0.11, 0.01
Very good			Ref			Ref			Ref			Ref
Excellent		−0.01	−0.07, 0.06		0.04	−0.03, 0.11		−0.02	−0.09, 0.04		0.07	0.00, 0.13
Nausea/sickness—after 1st trimester	0.140			0.018			0.401			0.084		
No nausea		−0.05	−0.11, 0.01		−0.06	−0.12, 0.00		0.03	−0.03, 0.09		−0.05	−0.11, 0.01
Mild nausea (Nausea only)			Ref			Ref			Ref			Ref
Moderate Nausea (occasionally sick)		−0.09	−0.18, 0.00		−0.15	−0.24, −0.06		0.02	−0.07, 0.11		−0.10	−0.19, −0.01
Severe nausea (regularly sick, can’t hold meals)		0.01	−0.12, 0.14		−0.03	−0.17, 0.10		0.11	−0.02, 0.23		−0.13	−0.25, 0.00
EDPS ^1^	0.0005			0.138			0.284			0.102		
No (<13)			Ref			Ref			Ref			Ref
Yes (≥13)		0.14	0.06, 0.23		−0.06	−0.15, 0.02		−0.04	−0.12, 0.04		−0.07	−0.15, 0.01
Folic acid intake	0.0002			0.006			<0.0001			0.0002		
Never taken folic acid			Ref			Ref			Ref			Ref
Before pregnancy		−0.20	−0.30, −0.10		0.17	0.07, 0.28		−0.25	−0.35, −0.15		−0.12	−0.22, −0.02
After pregnancy		−0.18	−0.27,−0.09		0.12	0.03, 0.22		−0.13	−0.22, −0.04		−0.18	−0.27, −0.09
Pre/during pregnancy smoking patterns	0.0001			<0.0001			<0.0001			0.007		
Continued smoking		0.21	0.11, 0.31		−0.34	−0.45, −0.23		0.53	0.43, 0.63		−0.11	−0.21, −0.01
Stopped smoking		0.07	−0.02, 0.16		−0.07	−0.17, 0.02		0.14	0.05, 0.23		−0.13	−0.22, −0.03
Non-smokers			Ref			Ref			Ref			Ref
Pre/during pregnancy alcohol consumption	0.0003			0.646			0.008			0.109		
Any drinking during		0.13	0.06, 0.20		−0.01	−0.08, 0.07		−0.11	−0.18, −0.04		−0.06	−0.13, 0.02
Stopped drinking		0.12	0.06, 0.19		0.02	−0.05, 0.09		−0.07	−0.14, −0.01		−0.07	−0.14, 0.00
Non-drinkers			Ref			Ref			Ref			Ref
Pre/during pregnancy physical activity	0.414			<0.0001			0.004			0.0004		
Mod/vigorous activity during		0.03	−0.12, 0.19		0.11	−0.06, 0.27		0.03	−0.12, 0.19		0.10	−0.06, 0.26
No mod/vigorous activity during		0.01	−0.05, 0.07		0.13	0.06, 0.19		−0.11	−0.17, −0.05		0.09	0.03, 0.16
Mod/vigorous activity before & during		−0.04	−0.10, 0.02		0.16	0.10, 0.22		−0.01	−0.07, 0.04		0.12	0.06, 0.18
No mod/vigorous activity before & during			Ref			Ref			Ref			Ref
Actively dieting pre-pregnancy	<0.0001			0.050			<0.001			0.133		
Yes		0.14	0.08, 0.20		−0.06	−0.12, 0.00		−0.14	−0.20, −0.08		−0.05	−0.11, 0.01
No			Ref			Ref			Ref			Ref

^1^ EPDS score of 13 or greater indicates that a women is likely to be suffering from symptoms of depression [26]; BMI, body mass index; LMC, lead maternity carer; EPDS, Edinburgh Postnatal Depression Scale; ref, reference.

**Table 3:** Association of each maternal dietary pattern with adherence to dietary guideline recommendations for number of daily servings from each of the four main food groups, adjusting for all maternal socio-demographic, health and lifestyle factors ^1^ (Odds ratios (OR) and 95% confidence intervals (CI)).

	Junk			Health Conscious			Traditional/White Bread			Fusion/Protein		
	*p*	OR	95% CI	*p*	OR	95% CI	*p*	OR	95% CI	*p*	OR	95% CI
Vegetables and fruits (≥6 serves/day)	<0.0001			<0.0001			0.0005			<0.0001		
Yes		0.56	0.48, 0.65		6.37	5.29, 7.67		1.32	1.13, 1.54		4.79	4.05, 5.65
No		Ref			Ref			Ref			Ref	
Breads and Cereals (≥4 serves/day)	<0.0001			<0.0001			<0.0001			<0.0001		
Yes		2.56	2.21, 2.97		3.05	2.55, 3.65		2.89	2.48, 3.38		0.56	0.46, 0.67
No		Ref			Ref			Ref			Ref	
Milk and milk products (≥3 serves/day)	<0.0001			<0.0001			<0.0001			0.054		
Yes		1.34	1.18, 1.53		4.60	3.97, 5.32		2.00	1.74, 2.30		1.16	1.00, 1.35
No		Ref			Ref			Ref			Ref	
Lean meat, meat alternatives and eggs (≥2serves/day)	0.045			0.347			0.807			<0.0001		
Yes		1.19	1.00, 1.40		1.10	0.91, 1.33		1.02	0.86, 1.22		14.3	11.5, 17.8
No		Ref			Ref			Ref			Ref	

^1^ Adjusted for age, pre-pregnancy BMI, self-prioritised ethnicity, place of birth, education, socioeconomic deprivation, pregnancy planning, parity, LMC, season of interview, maternal self-rating of health, nausea/sickness after 1st trimester, Edinburgh Postnatal Depression Scale, folic acid intake, pre/during pregnancy smoking patterns, pre/during pregnancy alcohol consumption, physical activity, and actively dieting pre-pregnancy; ref, reference.

**Table 4:** Associations of each maternal dietary pattern with the number of food group guidelines for which adherence was present, adjusting for all maternal socio-demographic, health and lifestyle factors ^1^ (Odds ratios (OR) and 95% confidence intervals (CI)).

	Junk			Health Conscious			Traditional/White Bread			Fusion/Protein		
	*p*	OR	95% CI	*p*	OR	95% CI	*p*	OR	95% CI	*p*	OR	95% CI
No. of food group guidelines	<0.0001			<0.0001			<0.0001			<0.0001		
None		Ref			Ref			Ref			Ref	
One guideline		1.35	1.15, 1.59		4.81	4.00, 5.79		2.23	1.85, 2.67		2.00	1.67, 2.41
Two-four guidelines		1.66	1.41, 1.96		19.2	15.6, 23.6		3.99	3.32, 4.79		5.75	4.78, 6.92

^1^ Adjusted for age, pre-pregnancy BMI, self-prioritised ethnicity, place of birth, education, socioeconomic deprivation, pregnancy planning, parity, LMC, season of interview, maternal self-rating of health, nausea/sickness after 1st trimester, Edinburgh Postnatal Depression Scale, folic acid intake, pre/during pregnancy smoking patterns, pre/during pregnancy alcohol consumption, physical activity, and actively dieting pre-pregnancy; ref, reference.

## Supplementary Materials

The following are available online at http://www.mdpi.com/2072-6643/8/5/300/s1, Table S1: Factor loadings of various food items in the four dietary components obtained using principal component analysis; Table S2: Univariate/unadjusted association of each maternal dietary pattern with adherence to dietary guideline recommendations for number of daily servings from each of the four main food groups.

## Abbreviations

The following abbreviations are used in this manuscript:

ALSPAC	Avon Longitudinal Study of Parents and Children
BMI	body mass index
DNBC	Danish National Birth Cohort
DIPP	Finnish Type 1 Diabetes Prediction and Prevention
FFQ	food frequency questionnaire
LMC	lead maternity carer
NMC	Norwegian Mother and Child
NZ	New Zealand
PCA	principal components analysis
