# Supplementary material for: Dietary Patterns in Pregnancy in New Zealand—Influence of Maternal Socio-Demographic, Health and Lifestyle Factors
Source: Nutrients. 2016 May 19;8(5):300. doi: 10.3390/nu8050300 (PMC4882712; doi:10.3390/nu8050300)
Supplement: Supplementary file 1 [file nutrients-08-00300-s001.docx]

Dietary Patterns in Pregnancy in New Zealand—Influence of Maternal Socio-Demographic, Health and Lifestyle Factors

Clare R. Wall, Cheryl S. Gammon, Dinusha K. Bandara, Cameron C. Grant, Polly E. Atatoa Carr and Susan M.B. Morton

**Table S1.** Factor loadings of various food items in the four dietary components obtained using principal component analysis ^1^.

| **Food items from FFQ** | **Junk** | **Health conscious** | **Traditional/ White bread** | **Fusion/Protein** |
| --- | --- | --- | --- | --- |
| Dietary pattern (variance explained) | **(7.7%)** | **(5.6%)** | **(5.4%)** | **(4.8%)** |
| Confectionary | **0.654** | 0.109 | 0.038 | −0.096 |
| Snacks—crisps, nuts | **0.644** | 0.087 | 0.078 | 0.088 |
| Takeaways | **0.636** | −0.126 | 0.084 | 0.116 |
| Hot chips | **0.571** | −0.062 | 0.181 | 0.122 |
| Processed meat | **0.488** | −0.051 | 0.134 | 0.232 |
| Soft drinks, energy drinks | **0.487** | −0.175 | 0.215 | −0.095 |
| Battered, fried fish, seafood | **0.479** | −0.050 | 0.073 | 0.256 |
| Ice cream | **0.461** | 0.0411 | 0.128 | 0.132 |
| Cakes or biscuits | **0.449** | 0.212 | 0.048 | −0.080 |
| Fruit juices | 0.240 | 0.146 | 0.215 | 0.103 |
| Soft drinks sugar free | 0.180 | 0.101 | −0.124 | −0.025 |
| Other vegetables | −0.067 | **0.543** | −0.025 | 0.153 |
| Cheese | 0.123 | **0.531** | −0.022 | −0.058 |
| Brown wholemeal bread | −0.032 | **0.523** | 0.018 | −0.139 |
| Non-citrus fruits | −0.021 | **0.464** | 0.035 | 0.188 |
| Yoghurt | 0.067 | **0.449** | 0.043 | 0.143 |
| Dried fruits | −0.082 | **0.426** | −0.048 | 0.143 |
| High fiber cereal | 0.032 | **0.392** | 0.011 | 0.037 |
| Vegemite™, Marmite™ | 0.185 | **0.345** | **0.307** | −0.193 |
| Lite or reduced fat margarine | 0.078 | 0.247 | −0.236 | −0.124 |
| Reduced fat milk | 0.120 | 0.214 | −0.273 | −0.149 |
| Skim or trim milk | 0.025 | 0.192 | −0.361 | 0.058 |
| Other bread | −0.013 | 0.138 | −0.0754 | 0.078 |
| Plant sterol margarine | 0.001 | 0.0303 | −0.044 | −0.005 |
| Whole or standard milk | 0.029 | −0.076 | **0.726** | 0.058 |
| White bread | **0.308** | −0.227 | **0.576** | 0.028 |
| Margarine | 0.129 | 0.077 | **0.450** | −0.186 |
| Jam honey marmalade | 0.141 | 0.278 | **0.434** | −0.011 |
| Peanut butter, Nutella™ | 0.224 | 0.179 | **0.337** | −0.014 |
| Other cereal ^2^ | 0.196 | −0.073 | **0.316** | 0.093 |
| Butter | 0.093 | 0.0612 | 0.277 | 0.152 |
| Butter and margarine blend | 0.120 | −0.005 | 0.220 | −0.057 |
| High fiber white bread | 0.033 | 0.036 | 0.131 | 0.088 |
| Noodles rice pasta | 0.042 | −0.052 | 0.055 | **0.527** |
| Seafood | 0.1237 | 0.007 | −0.017 | **0.511** |
| Chicken | 0.316 | −0.134 | 0.039 | **0.504** |
| Green leafy vegetables | −0.062 | **0.307** | 0.112 | **0.461** |
| Eggs | 0.137 | 0.052 | 0.241 | **0.427** |
| Red meat | 0.214 | −0.007 | 0.217 | **0.395** |
| Citrus fruits | −0.012 | 0.167 | 0.255 | 0.277 |
| Processed fish | 0.133 | 0.134 | −0.054 | 0.273 |
| Alternative protein, nuts, tofu | −0.164 | 0.239 | −0.089 | 0.254 |
| Soy milk | −0.159 | 0.088 | −0.048 | 0.222 |
| Other milk | 0.001 | 0.056 | −0.054 | 0.101 |

^1^ Loadings above 0.3 are shown in bold; ^2^ low fibre and/or high sugar cereals; FFQ: food frequency questionnaire.

**Table S2.** Univariate/unadjusted association of each maternal dietary pattern with adherence to dietary guideline recommendations for number of daily servings from each of the four main food groups**.**

|  | **Junk** | | | **Health conscious** | | | **Traditional/White bread** | | | **Fusion/Protein** | | |
| --- | --- | --- | --- | --- | --- | --- | --- | --- | --- | --- | --- | --- |
|  | *P* | OR | 95% CI | *P* | OR | 95% CI | *P* | OR | 95% CI | *P* | OR | 95% CI |
| Vegetables and fruits  (≥6 serves/day) | <0.0001 |  |  | <0.0001 |  |  | 0.002 |  |  | <0.0001 |  |  |
| Yes |  | 0.63 | 0.56, 0.72 |  | 5.37 | 4.67, 6.18 |  | 1.22 | 1.08, 1.38 |  | 4.20 | 3.69, 4.78 |
| No |  | Ref |  |  | Ref |  |  | Ref |  |  | Ref |  |
| Breads and Cereals  (≥4 serves/day) | <0.0001 |  |  | <0.0001 |  |  | <0.0001 |  |  | 0.888 |  |  |
| Yes |  | 2.64 | 2.34, 2.98 |  | 1.94 | 1.72, 2.19 |  | 4.21 | 3.71, 4.77 |  | 1.01 | 0.89, 1.14 |
| No |  | Ref |  |  | Ref |  |  | Ref |  |  | Ref |  |
| Milk and milk products  (≥3 serves/day) | <0.0001 |  |  | <0.0001 |  |  | <0.0001 |  |  | 0.0001 |  |  |
| Yes |  | 1.46 | 1.31, 1.62 |  | 4.56 | 4.07, 5.11 |  | 1.80 | 1.62, 2.01 |  | 1.20 | 1.08, 1.34 |
| No |  | Ref |  |  | Ref |  |  | Ref |  |  | Ref |  |
| Lean meat, meat alternatives and eggs (≥2 serves/day) | 0.002 |  |  | 0.509 |  |  | <0.0001 |  |  | <0.0001 |  |  |
| Yes |  | 1.22 | 1.08, 1.39 |  | 1.04 | 0.92, 1.18 |  | 1.77 | 1.56, 2.01 |  | 13.77 | 11.63, 16.30 |
| No |  | Ref |  |  | Ref |  |  | Ref |  |  | Ref |  |
